# Discovery of talmapimod analogues as polypharmacological anti-inflammatory agents

**DOI:** 10.1080/14756366.2019.1693703

**Published:** 2019-11-22

**Authors:** Wandong Liu, Caiyun Hou, Jiaming Li, Xiaodong Ma, Yanchun Zhang, Mengqi Hu, Yuanzheng Huang

**Affiliations:** a School of Pharmacy, Anhui University of Chinese Medicine, Hefei, China; b Department of Medicinal Chemistry, Anhui Academy of Chinese Medicine, Hefei, China

**Keywords:** Polypharmacological agent, anti-inflammation, talmapimod analogues, p38α MAPK, COXs

## Abstract

Twenty novel talmapimod analogues were designed, synthesised and evaluated for the *in vivo* anti-inflammatory activities. Among them, compound **6n**, the most potent one, was selected for exploring the mechanisms underlying its anti-inflammatory efficacy. In RAW264.7 cells, it effectively suppressed lipopolysaccharides-induced (LPS-induced) expressions of iNOS and COX-2. As illustrated by the western blot analysis, **6n** downregulated both the NF-κB signalling and p38 MAPK phosphorylation. Further enzymatic assay identified **6n** as a potent inhibitor against both p38α MAPK (IC_50_=1.95 µM) and COX-2 (IC_50_=0.036 µM). By virtue of the concomitant inhibition of p38α MAPK, its upstream effector, and COX-2, along with its capability to downregulate NF-κB and MAPK-signalling pathways, **6n**, a polypharmacological anti-inflammatory agent, deserves further development as a novel anti-inflammatory drug.

## 1. Introduction

So far, inflammatory diseases, especially the chronic inflammatory disorders, have continuously to be a major global health concern due to the lack of effective and well-tolerated drugs^1–3^. Currently, the majority of anti-inflammatory therapies have been focussed on two distinct strategies, the first directly interferes with the biological function of the pro-inflammatory mediators by interacting with them or their targets, and the second blocks the production of pro-inflammatory mediators^4^. However, given the complex mechanism underlying some inflammatory diseases, which are involved with multiple signalling pathways, the legendary magic bullet, a drug with high potency and selectivity towards a specific biological target, is insufficient for curing them^4–7^. Two approaches are capable of achieving multi-dimensional regulation of disease-related signalling pathways, including the drug combination and polypharmacology featuring simultaneous modulation of multiple targets with a single drug molecule^8^^,^^9^. Between them, the latter benefits from the potential to obviate the drug–drug interactions and minimise the combined off-target effects^10–13^.

**Figure 1.**
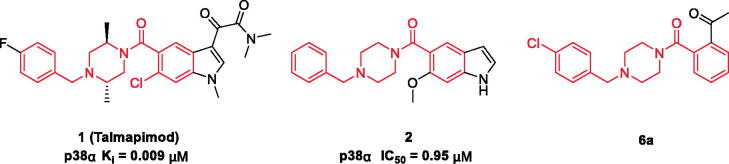
Structures and potencies of **1**, **2** and talmapimod analogue **6a**.

**Figure 2.**
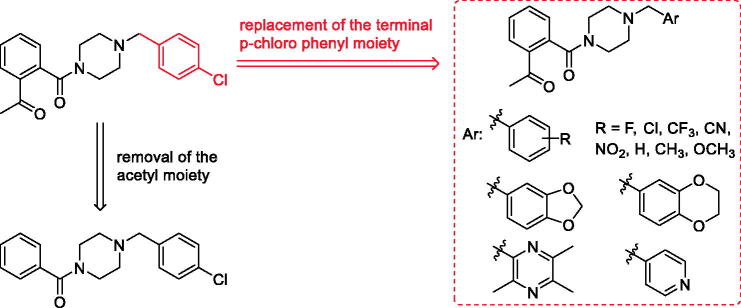
The design of talmapimod analogues.

Talmapimod **1**, as a highly selective p38α mitogen-activated protein kinase (p38α MAPK) inhibitor developed by Scios. Inc. from compound **2**^14^, has been advanced to Phase-II clinical trials for the treatment of rheumatoid arthritis, multiple myeloma and bone marrow diseases^15^. From an internal programme to prepare butylphthalide derivatives, an undesired compound **6a** was obtained via the previously designed synthetic route. Owing to its structural similarity to **1** and **2**, we are intrigued by the potential of **6a** and its derivatives as anti-inflammatory agents (Figure 1). Hence, on the basis of **6a**, a series of talmapimod analogues were designed and synthesised as shown in Figure 2. With the attempt to validate their anti-inflammatory efficacy, these talmapimod analogues were first evaluated *in vivo*. The most potent compound **6n** was further tested for the inhibitory activity against nitric oxide (NO) production in RAW264.7 cells, and its anti-inflammatory mechanism was investigated by western blot. Additionally, according to the results of mechanism study, compounds with promising anti-inflammatory activity *in vivo* were selected for evaluating the p38α MAPK and cyclooxygenases (COXs) inhibitory activities. Finally, molecular docking studies were conducted to elucidate the possible binding modes with these proteins.

## 2. Experimental

### 2.1. Chemistry

Starting materials, reagents and solvents were purchased from common commercial suppliers. If necessary, purification was carried out prior to use. Melting points were uncorrected and determined on a WRS-1B apparatus. ^1^H and ^13 ^C NMR spectra were recorded on Bruker Avance 400 II (400 MHz) spectrometer using DMSO-*d_6_* with tetramethylsilane (TMS) as internal standard. ESI-MS were obtained by Thermo Q-Exactive spectrometer.

#### 2.1.1. General procedure for target compounds 6a-6s

3-(Iodomethyl)-3H-isobenzofuran-1-one (**4**). Iodine (9.0 g, 36 mmol) was added in a solution of 2-vinylbenzoic acid (2.7 g, 18 mmol) in CH_3_CN (30 ml). The reaction mixture was stirred at 25 °C for 1 h under N_2_ atmosphere and quenched with saturated Na_2_S_2_O_3_ solution. The mixture was extracted with EA. The EA layer phase was washed successively with water, NaHCO_3_, Na_2_S_2_O_3_, dried over Na_2_SO_4_ and concentrated to a yellow solid. The crude product was purified by recrystallization from hot ethanol, afforded the title compound as a white crystal, Yield: 43%; m.p. 86.9 – 88.4 °C; ^1^H NMR (400 MHz, DMSO-*d*_6_) δ: 7.92 – 7.79 (*m*, 2H, Ar-**H**), 7.74 (d, 1H *J* = 7.7, Ar-**H**), 7.69 – 7.61 (*m*, 1H, Ar-**H**), 5.66 (*t*, 1H, *J* = 4.0 Hz, C**H**), 3.97 (dd, 1H, *J* = 11.3, 3.9 Hz, C**H**), 3.87 (dd, 1H, *J* = 11.3, 4.3 Hz, C**H**). ESI-Mass for C_9_H_7_IO_2_ *m/z*: 274.7 [M + H]^+^.

1–(2-(4–(4-Chlorobenzyl)piperazine-1-carbonyl)phenyl)ethan-1-one (**6a**) A solution of **4** (690 mg, 3.0 mmol) in DCM (10.0 ml) was added dropwise to a solution of 1–(4-chlorobenzyl)piperazine (840 mg, 4.0 mmol) and K_2_CO_3_ (700 mg, 5.0 mmol) in 20 ml DCM. The reaction mixture was stirred at 25 °C for 3 h. The mixture was washed successively with H_2_O, brine, dried over Na_2_SO_4_ and concentrated *in vacuo*. The residue was purified by flash column chromatography utilising PE/EA (2:1) as the eluent to afforded the title compound as white solid, Yield: 35%; m.p. 97.5 – 98.4 °C; ^1^H NMR (400 MHz, DMSO-*d*_6_) δ: 7.98 (dd, 1H, *J* = 7.7, 1.2 Hz, Ar-**H**), 7.63 (dd, 1H, *J* = 7.5, 1.3 Hz, Ar-**H**), 7.55 (dd, 1H, *J* = 7.5, 1.3 Hz, Ar-**H**), 7.41 – 7.36(*m*, 2H, Ar-**H**), 7.36 – 7.31 (*m*, 2H, Ar-**H**), 7.29 (dd, 1H, *J* = 7.7, 1.2 Hz, Ar-**H**), 3.59 (*t*, 2H, *J* = 5.1 Hz, piperazine-**H**), 3.42 (*s*, 2H, C**H_2_**), 3.04 (*t*, 2H, *J* = 5.0 Hz, piperazine-**H**), 2.55 (*s*, 3H, C**H_3_**), 2.44 (*t*, 2H, *J* = 5.1 Hz, piperazine-**H**), 2.28 (*t*, 2H, *J* = 5.0 Hz, piperazine-**H**); ^13 ^C NMR (100 MHz, DMSO-*d_6_*) δ: 198.57 (C-6, C = O, ketone), 170.28 (C-9, C = O, amide), 137.19 (C-5), 136.46 (C-15), 135.31 (C-8), 132.86 (C-2), 132.62 (C-18), 130.31 (C-3), 129.66 (C-16, 20), 128.85 (C-17, 19), 128.45(C-4), 127.24 (C-1), 62.04 (C-14, –CH_2_), 52.50 (C-11, –CH_2_, Piperazine), 52.34 (C-12, –CH_2_, Piperazine), 46.83 (C-13, –CH_2_, Piperazine), 41.62 (C-10, –CH_2_, Piperazine), 27.67 (C-7, –CH_3_). ESI-Mass for C_20_H_21_ClN_2_O_2_ *m/z*: 357.229 [M + H]^+^.

Compound **6 b**-**6s** were prepared in a procedure similar to that described for **6a**.

1–(2-(4–(4-Methoxybenzyl)piperazine-1-carbonyl)phenyl)ethan-1-one (**6 b**) white solid; Yield: 36%; m.p. 88.4–89.6 °C; ^1^H NMR (400 MHz, DMSO-*d*_6_) δ: 7.98 (dd, *J* = 7.7, 1.2 Hz, 1H, Ar-**H**), 7.63 (d, *J* = 7.5, 1.3 Hz, 1H, Ar-**H**), 7.55 (d, *J* = 7.5, 1.4 Hz, 1H, Ar-**H**), 7.32 – 7.25 (*m*, 1H, Ar-**H**), 7.25 – 7.18 (*m*, 2H, Ar-**H**), 6.94 – 6.84 (*m*, 2H, Ar-**H**), 3.73 (*s*, 3H, C**H_3_**), 3.57 (d, *J* = 5.1 Hz, 2H, piperazine-**H**), 3.42 (*s*, 2H, C**H_2_**), 3.04 (*t*, *J* = 5.0 Hz, 2H, piperazine-**H**), 2.55 (*s*, 3H, C**H_3_**), 2.41 (*t*, *J* = 5.1 Hz, 2H, piperazine-**H**), 2.26 (*t*, *J* = 5.0 Hz, 2H, piperazine-**H**). ^13 ^C NMR (100 MHz, DMSO-*d_6_*) δ: 199.22 (C-6, C = O, ketone), 169.35 (C-9, C = O, amide), 158.78 (C-18), 137.12 (C-5), 135.80 (C-8), 132.92 (C-2), 130.54 (C-16, 20), 130.25 (C-3), 130.12 (C-15), 129.28 (C-4), 127.41 (C-1), 114.04 (C-17, 19), 61.78 (C-14, –CH_2_), 55.45 (C-21, –OCH_3_), 52.47 (C-11, –CH_2_, Piperazine), 52.16 (C-12, –CH_2_, Piperazine), 46.75 (C-13, –CH_2_, Piperazine), 41.54 (C-10, –CH_2_, Piperazine), 28.33 (C-7, –CH_3_). ESI-Mass for C_20_H_24_N_2_O_3_ *m/z*: 353.232 [M + H]^+^.

4-((4–(2-Acetylbenzoyl)piperazin-1-yl)methyl)benzonitrile (**6c**) white solid; Yield: 46%; m.p. 143.6–145.6 °C; ^1^H NMR (400 MHz, DMSO-*d_6_*) δ: 7.98 (dd, *J =* 7.8, 1.2 Hz, 1H, Ar-**H**), 7.80 (d, *J =* 8.2 Hz, 2H, Ar-**H**), 7.64 (dd, *J =* 7.5, 1.3 Hz, 1H, Ar-**H**), 7.59 – 7.49 (*m*, 3H, Ar-**H**), 7.29 (dd, *J =* 7.8, 1.2 Hz, 1H, Ar-**H**), 3.65 – 3.57 (*m*, 4H, piperazine-H, C**H_2_**), 3.06 (*t*, *J =* 5.0 Hz, 2H, piperazine-**H**), 2.56 (s, 3H, C**H_3_**), 2.46 (*t*, *J =* 5.1 Hz, 2H, piperazine-**H**), 2.30 (*t*, *J =* 5.0 Hz, 2H, piperazine-**H**). ^13 ^C NMR (100 MHz, DMSO-*d_6_*) δ 199.22 (C-6, C = O, ketone), 169.41 (C-9, C = O, amide), 144.61 (C-15), 137.07 (C-5), 135.70 (C-8), 132.98 (C-2), 132.65 (C-16, 20), 130.33 (C-3), 130.01 (C-17, 19), 129.32 (C-4), 127.40 (C-1), 119.36 (C-21), 110.23 (C-18), 61.60 (C-14, –CH_2_), 52.53 (C-11, –CH_2_, Piperazine), 52.29 (C-12, –CH_2_, Piperazine), 46.70 (C-13, –CH_2_, Piperazine), 41.50 (C-10, –CH_2_, Piperazine), 28.31 (C-7, –CH_3_). ESI-Mass for C_20_H_21_N_3_O_2_ *m/z*: 348.237 [M + H]^+^.

1–(2-(4–(4-(Trifluoromethyl)benzyl)piperazine-1-carbonyl)phenyl)ethan-1-one (**6d**) white solid; Yield: 31%; m.p. 63.1–64.5 °C; ^1^H NMR (400 MHz, DMSO-*d_6_*) δ: 7.99 (dd, *J =* 7.7, 1.3 Hz, 1H, Ar-**H**), 7.69 (d, *J =* 8.1 Hz, 2H, Ar-**H**), 7.64 (dd, *J =* 7.5, 1.3 Hz, 1H, Ar-**H**), 7.60 – 7.51 (*m*, 3H, Ar-**H**), 7.29 (dd, *J =* 7.7, 1.3 Hz, 1H, Ar-**H**), 3.66 – 3.57 (*m*, 4H, piperazine-**H**, C**H_2_**), 3.07 (dd, *J =* 5.0, 4.1 Hz, 2H, piperazine-**H**), 2.56 (*s*, 3H, C**H_3_**), 2.46 (d, *J =* 5.0 Hz, 2H, piperazine-**H**), 2.31 (*t*, *J =* 5.0 Hz, 2H, piperazine-**H**). ^13 ^C NMR (100 MHz, DMSO-*d*_6_) δ: 199.20 (C-6, C=O, ketone), 169.41 (C-9, C=O, amide), 143.51 (C-15), 137.09 (C-5), 135.71 (C-8), 132.96 (C-2), 130.31 (C-3), 129.84 (C-16, 20), 129.30 (C-4), 128.12 (*J*_C-F_ = 31.6 Hz) (C-18), 127.40 (C-1), 125.53 (*J*_C-F_ = 3.8 Hz) (C-17, 19), 124.79 (*J*_C-F_ = 270.0 Hz) (C-21), 61.58 (C-14, –CH_2_), 52.55 (C-11, –CH_2_, Piperazine), 52.28 (C-12, –CH_2_, Piperazine), 46.70 (C-13, –CH_2_, Piperazine), 41.51 (C-10, –CH_2_, Piperazine), 28.29 (C-7, –CH_3_). ESI-Mass for C_21_H_21_F_3_N_2_O_2_ *m/z*: 391.251 [M + H]^+^.

1–(2-(4–(4-Nitrobenzyl)piperazine-1-carbonyl)phenyl)ethan-1-one (**6e**) white solid; Yield: 42%; m.p. 190.8–192.4 °C; ^1^H NMR (400 MHz, DMSO-*d_6_*) δ: 8.29 – 8.15 (*m*, 2H, Ar-**H**), 7.98 (dd, *J =* 7.8, 1.3 Hz, 1H, Ar-**H**), 7.69 – 7.59 (*m*, 3H, Ar-**H**), 7.55 (dd, *J =* 7.5, 1.3 Hz, 1H, Ar-**H**), 7.29 (dd, *J =* 7.8, 1.3 Hz, 1H, Ar-**H**), 3.65 (*s*, 2H, C**H_2_**), 3.64 – 3.56 (*m*, 2H, piperazine-**H**), 3.07 (dd, *J =* 5.0, 4.0 Hz, 2H, piperazine-**H**), 2.55 (*s*, 3H, C**H_3_**), 2.48 (*t*, *J =* 5.0 Hz, 2H, piperazine-**H**), 2.32 (*t*, *J =* 5.0 Hz, 2H, piperazine-**H**). ^13 ^C NMR (100 MHz, DMSO-*d_6_*) δ: 199.24 (C-6, C=O, ketone), 169.42 (C-9, C=O, amide), 147.05 (C-15), 146.91 (C-18), 137.06 (C-5), 135.70 (C-8), 132.99 (C-2), 130.33 (C-3), 130.18 (C-16, 20), 129.33 (C-4), 127.40 (C-1), 123.86 (C-17, 19), 61.28 (C-14, –CH_2_), 52.55 (C-11, –CH_2_, Piperazine), 52.31 (C-12, –CH_2_, Piperazine), 46.70 (C-13, –CH_2_, Piperazine), 41.50 (C-10, –CH_2_, Piperazine), 28.32 (C-7, –CH_3_). ESI-Mass for C_20_H_21_N_3_O_4_ *m/z*: 368.240 [M + H]^+^.

1–(2-(4–(3-Nitrobenzyl)piperazine-1-carbonyl)phenyl)ethan-1-one (**6f**) white solid; Yield: 30%; m.p. 180.1–181.8 °C; ^1^H NMR (400 MHz, DMSO-*d_6_*) δ: 8.18 (*t*, *J =* 1.9 Hz, 1H, Ar-**H**), 8.15 – 8.10 (m, 1H, Ar-**H**), 7.98 (dd, *J =* 7.7, 1.3 Hz, 1H, Ar-**H**), 7.79 (dd, *J =* 7.6, 1.2 Hz, 1H, Ar-**H**), 7.67 – 7.61 (*m*, 2H, Ar-**H**), 7.55 (dd, *J =* 7.5, 1.2 Hz, 1H, Ar-**H**), 7.29 (dd, *J =* 7.7, 1.3 Hz, 1H, Ar-**H**), 3.65 (*s*, 2H, C**H_2_**), 3.61 (d, *J =* 5.0 Hz, 2H, piperazine-**H**), 3.07 (dd, *J =* 5.0, 4.0 Hz, 2H, piperazine-**H**), 2.56 (*s*, 3H, C**H_3_**), 2.48 (d, *J* = 5.0 Hz, 2H, piperazine-**H**), 2.38 – 2.28 (*m*, 2H, piperazine-**H**). ^13 ^C NMR (100 MHz, DMSO-*d_6_*) δ: 199.25 (C-6, C=O, ketone), 169.40 (C-9, C=O, amide), 148.29 (C-17), 137.06 (C-5), 135.95 (C-15), 135.70 (C-8), 132.98 (C-2), 130.33 (C-3), 130.24 (C-20), 129.32 (C-4), 127.40 (C-1), 123.60 (C-16), 122.55 (C-18), 61.02 (C-14, –CH_2_), 52.42 (C-11, –CH_2_, Piperazine), 52.19 (C-12, –CH_2_, Piperazine), 46.70 (C-13, –CH_2_, Piperazine), 41.48 (C-10, –CH_2_, Piperazine), 28.32 (C-7, –CH_3_). ESI-Mass for C_20_H_21_N_3_O_4_ *m/z*: 368.253 [M + H]^+^.

1–(2-(4–(2,4-Dichlorobenzyl)piperazine-1-carbonyl)phenyl)ethan-1-one (**6 g**) white solid; Yield: 37%; m.p. 74.9–76.4 °C; ^1^H NMR (400 MHz, DMSO-*d_6_*) δ: 7.99 (dd, *J* = 7.8, 1.3 Hz, 1H, Ar-**H**), 7.66 – 7.62 (*m*, 1H, Ar-**H**), 7.60 (d, *J* = 2.2 Hz, 1H, Ar-**H**), 7.58 – 7.50 (*m*, 2H, Ar-**H**), 7.42 (dd, *J* = 8.3, 2.2 Hz, 1H, Ar-**H**), 7.29 (dd, *J* = 7.8, 1.3 Hz, 1H, Ar-**H**), 3.60 (*t*, *J* = 5.0 Hz, 2H, piperazine-**H**), 3.58 (*s*, 2H, C**H_2_**), 3.10 – 3.02 (*m*, 2H, piperazine-**H**), 2.56 (*s*, 3H, C**H_3_**), 2.49 (d, *J =* 5.0 Hz, 2H, piperazine-**H**), 2.34 (*t*, *J =* 5.0 Hz, 2H, piperazine-**H**). ^13 ^C NMR (100 MHz, DMSO-*d_6_*) δ: 199.24 (C-6, C=O, ketone), 169.40 (C-9, C=O, amide), 137.07 (C-5), 135.73 (C-8), 135.06 (C-15), 134.63 (C-16), 132.98 (C-2), 132.72 (C-18), 132.56 (C-20), 130.32 (C-3), 129.32 (C-4), 129.16 (C-17), 127.70 (C-19), 127.41 (C-1), 58.33 (C-14, –CH_2_), 52.54 (C-11, –CH_2_, Piperazine), 52.34 (C-12, –CH_2_, Piperazine), 46.72 (C-13, –CH_2_, Piperazine), 41.52 (C-10, –CH_2_, Piperazine), 28.33 (C-7, –CH_3_). ESI-Mass for C_20_H_20_ClN_2_O_2_ *m/z*: 391.183 [M + H]^+^.

1–(2-(4–(2-Chlorobenzyl)piperazine-1-carbonyl)phenyl)ethan-1-one (**6 h**) white solid; Yield: 44%; m.p. 133.5–134.6 °C; ^1^H NMR (400 MHz, DMSO-*d_6_*) δ: 7.99 (dd, *J =* 7.8, 1.3 Hz, 1H, Ar-**H**), 7.66 – 7.62 (*m*, 1H, Ar-**H**), 7.59 – 7.48 (*m*, 2H, Ar-**H**), 7.43 (dd, *J* = 7.7, 1.6 Hz, 1H, Ar-**H**), 7.37 – 7.26 (*m*, 3H, Ar-**H**), 3.60 (d, *J* = 5.1 Hz, 4H, piperazine-H, C**H_2_**), 3.06 (dd, *J* = 5.0, 4.0 Hz, 2H, piperazine-**H**), 2.56 (*s*, 3H, C**H_3_**), 2.49 (d, *J* = 5.0 Hz, 2H, piperazine-**H**), 2.35 (*t*, *J* = 5.0 Hz, 2H, piperazine-**H**). ^13 ^C NMR (100 MHz, DMSO-*d_6_*) δ: 199.25 (C-6, C=O, ketone), 169.39 (C-9, C=O, amide), 137.09 (C-5), 135.76 (C-8), 135.73 (C-15), 133.77 (C-16), 132.98 (C-2), 131.33 (C-20), 130.32 (C-3), 129.74 (C-18), 129.31 (C-4), 129.17 (C-17), 127.52 (C-19), 127.42 (C-1), 58.96 (C-14, –CH_2_), 52.63 (C-11, –CH_2_, Piperazine), 52.40 (C-12, –CH_2_, Piperazine), 46.73 (C-13, –CH_2_, Piperazine), 41.54 (C-10, –CH_2_, Piperazine), 28.33 (C-7, –CH_3_). ESI-Mass for C_20_H_21_ClN_2_O_2_ *m/z*: 357.229 [M + H]^+^.

1–(2-(4–(3-Chlorobenzyl)piperazine-1-carbonyl)phenyl)ethan-1-one (**6i**) white solid; Yield: 48%; m.p. 105.8–107.5 °C;^1^H NMR (400 MHz, DMSO-*d_6_*) δ: 7.99 (dd, *J* = 7.8, 1.3 Hz, 1H, Ar-**H**), 7.66 – 7.62 (*m*, 1H, Ar-**H**), 7.55 (dd, *J* = 7.5, 1.3 Hz, 1H, Ar-**H**), 7.41 – 7.25 (*m*, 5H, Ar-**H**), 3.60 (*t*, *J* = 5.0 Hz, 2H, piperazine-**H**), 3.51 (*s*, 2H, C**H_2_**), 3.13 – 2.99 (*m*, 2H, piperazine-**H**), 2.55 (*s*, 3H, CH3), 2.45 (*t*, *J* = 5.0 Hz, 2H, piperazine-**H**), 2.29 (*t*, *J* = 5.0 Hz, 2H, piperazine-**H**). ^13 ^C NMR (100 MHz, DMSO-*d_6_*) δ: 199.20 (C-6, C=O, ketone), 169.38 (C-9, C=O, amide), 141.15 (C-15), 137.10 (C-5), 135.72 (C-8), 133.42 (C-17), 132.96 (C-2), 130.55 (C-19), 130.30 (C-3), 129.29 (C-4), 128.88 (C-16), 127.87 (C-18), 127.45 (C-1), 127.40 (C-20), 61.48 (C-14, –CH_2_), 52.47 (C-11, –CH_2_, Piperazine), 52.24 (C-12, –CH_2_, Piperazine), 46.71 (C-13, –CH_2_, Piperazine), 41.50 (C-10, –CH_2_, Piperazine), 28.32 (C-7, –CH_3_). ESI-Mass for C_20_H_21_ClN_2_O_2_ *m/z*: 357.212 [M + H]^+^.

1–(2-(4–(4-Fluorobenzyl)piperazine-1-carbonyl)phenyl)ethan-1-one (**6j**) white solid; Yield: 38%; m.p. 107.6–108.8 °C; ^1^H NMR (400 MHz, DMSO-*d_6_*) δ: 7.99 (dd, *J* = 7.8, 1.3 Hz, 1H, Ar-**H**), 7.63 (dd, *J* = 7.6, 1.3 Hz, 1H, Ar-**H**), 7.55 (dd, *J* = 7.6, 1.3 Hz, 1H, Ar-**H**), 7.40 – 7.31 (*m*, 2H, Ar-**H**), 7.28 (dd, *J* = 7.8, 1.3 Hz, 1H, Ar-**H**), 7.20 – 7.10 (*m*, 2H, Ar-**H**), 3.59 (*t*, *J* = 5.0 Hz, 2H, piperazine-**H**), 3.48 (*s*, 2H, C**H_2_**), 3.04 (*t*, *J* = 5.0 Hz, 2H, piperazine-**H**), 2.55 (*s*, 3H, C**H_3_**), 2.43 (*t*, *J* = 5.0 Hz, 2H, piperazine-**H**), 2.27 (*t*, *J* = 5.0 Hz, 2H, piperazine-**H**). ^13 ^C NMR (100 MHz, DMSO-*d_6_*) δ: 199.23 (C-6, C=O, ketone), 169.37 (C-9, C=O, amide), 161.73 (*J*_C-F_ = 242.5 Hz) (C-18), 137.09 (C-5), 135.74 (C-8), 134.51 (*J*_C-F_ = 2.9 Hz) (C-15), 132.96 (C-2), 131.13 (*J*_C-F_ = 8.1 Hz) (C-16, 20), 130.30 (C-3), 129.22 (C-4), 127.70 (C-1), 115.38 (*J*_C-F_ = 21.0 Hz) (C-17, 19), 61.40 (C-14, –CH_2_), 52.49 (C-11, –CH_2_, Piperazine), 52.17 (C-12, –CH_2_, Piperazine), 46.72 (C-13, –CH_2_, Piperazine), 41.51 (C-10, –CH_2_, Piperazine), 28.32 (C-7, –CH_3_). ESI-Mass for C_20_H_21_FN_2_O_2_ *m/z*: 341.199 [M + H]^+^.

1–(2-(4-Benzylpiperazine-1-carbonyl)phenyl)ethan-1-one (**6k**) white solid; Yield: 38%; m.p. 69.7–70.9 °C. ^1^H NMR (400 MHz, DMSO-*d_6_*) δ: 7.98 (dd, *J* = 7.8, 1.3 Hz, 1H, Ar-**H**), 7.63 (dd, *J* = 7.6, 1.3 Hz, 1H, Ar-**H**), 7.55 (dd, *J* = 7.6, 1.3 Hz, 1H, Ar-**H**), 7.36 – 7.22 (*m*, 6H, Ar-**H**), 3.59 (*t*, *J* = 5.0 Hz, 2H, piperazine-**H**), 3.50 (*s*, 2H, C**H_2_**), 3.05 (dd, *J* = 5.0, 4.1 Hz, 2H, piperazine-**H**), 2.55 (*s*, 3H, C**H_3_**), 2.44 (*t*, *J* = 5.0 Hz, 2H, piperazine-**H**), 2.29 (*t*, *J* = 5.0 Hz, 2H, piperazine-**H**). ^13 ^C NMR (100 MHz, DMSO-*d_6_*) δ: 199.21 (C-6, C=O, ketone), 169.36 (C-9, C=O, amide), 138.36 (C-15), 137.12 (C-5), 135.76 (C-8), 132.94 (C-2), 130.28 (C-3), 129.29 (C-4), 128.67 (C-16, 17, 19, 20), 127.47 (C-1), 127.41 (C-18), 62.38 (C-14, –CH_2_), 52.58 (C-11, –CH_2_, Piperazine), 52.29 (C-12, –CH_2_, Piperazine), 46.74 (C-13, –CH_2_, Piperazine), 41.53 (C-10, –CH_2_, Piperazine), 28.34 (C-7, –CH_3_). ESI-Mass for C_20_H_22_N_2_O_2_ *m/z*: 323.231 [M + H]^+^.

1–(2-(4–(4-Methylbenzyl)piperazine-1-carbonyl)phenyl)ethan-1-one (**6 l**) white solid; Yield: 46%; m.p. 116.7–118.3 °C.^1^H NMR (400 MHz, DMSO-*d_6_*) δ: 7.98 (dd, *J* = 7.8, 1.3 Hz, 1H, Ar-**H**), 7.63 (dd, *J* = 7.6, 1.3 Hz, 1H, Ar-**H**), 7.55 (dd, J = 7.6, 1.3 Hz, 1H, Ar-**H**), 7.28 (dd, J = 7.8, 1.3 Hz, 1H, Ar-**H**), 7.19 (d, *J* = 8.0 Hz, 2H, Ar-**H**), 7.13 (d, *J* = 8.0 Hz, 2H, Ar-**H**), 3.58 (*t*, *J* = 5.0 Hz, 2H, piperazine-**H**), 3.44 (*s*, 2H, C**H_2_**), 3.04 (*t*, *J* = 5.0 Hz, 2H, piperazine-**H**), 2.55 (*s*, 3H, C**H_3_**), 2.42 (*t*, *J* = 5.0 Hz, 2H, piperazine-**H**), 2.28 (*s*, 5H, piperazine-**H**, C**H_3_**). ^13 ^C NMR (100 MHz, DMSO-*d_6_*) δ: 199.20 (C-6, C=O, ketone), 169.37 (C-9, C=O, amide), 137.13 (C-5), 136.49 (C-15), 135.75 (C-8), 135.22 (C-18), 132.94 (C-2), 130.29 (C-3), 129.29 (C-4, 17, 19), 129.24 (C-16, 20), 127.40 (C-1), 62.14 (C-14, –CH_2_), 52.52 (C-11, –CH_2_, Piperazine), 52.24 (C-12, –CH_2_, Piperazine), 46.73 (C-13, –CH_2_, Piperazine), 41.53 (C-10, –CH_2_, Piperazine), 28.32 (C-7, –CH_3_), 21.17 (C-21, –CH_3_). ESI-Mass for C_21_H_24_N_2_O_2_ *m/z*: 337.255 [M + H]^+^.

1–(2-(4–(2,4-Dimethoxybenzyl)piperazine-1-carbonyl)phenyl)ethan-1-one (**6 m**) white solid; Yield: 46%; m.p. 113.9–115.4 °C. ^1^H NMR (400 MHz, DMSO-*d_6_*) δ: 7.97 (dd, *J* = 7.8, 1.3 Hz, 1H, Ar-**H**), 7.63 (dd, *J* = 7.6, 1.3 Hz, 1H, Ar-**H**), 7.54 (dd, *J* = 7.6, 1.3 Hz, 1H, Ar-**H**), 7.27 (dd, *J* = 7.8, 1.3 Hz, 1H, Ar-**H**), 7.17 (d, *J* = 8.3 Hz, 1H, Ar-**H**), 6.53 (d, *J* = 2.4 Hz, 1H, Ar-**H**), 6.49 (dd, *J* = 8.2, 2.4 Hz, 1H, Ar-**H**), 3.75 (d, *J* = 3.0 Hz, 6H, OC**H_3_×2**), 3.57 (*t*, *J* = 5.0 Hz, 2H, piperazine-**H**), 3.41 (*s*, 2H, C**H_2_**), 3.03 (*t*, *J* = 5.0 Hz, 2H, piperazine-**H**), 2.55 (*s*, 3H, C**H_3_**), 2.43 (*t*, *J* = 5.0 Hz, 2H, piperazine-**H**), 2.27 (*t*, *J* = 5.0 Hz, 2H, piperazine-**H**). ^13 ^C NMR (100 MHz, DMSO-*d_6_*) δ: 199.22 (C-6, C=O, ketone), 169.32 (C-9, C=O, amide), 160.06 (C-16), 158.85 (C-18), 137.14 (C-5), 135.76 (C-8), 132.94 (C-2), 131.37 (C-20), 130.27 (C-3), 129.27 (C-4), 127.41 (C-1), 117.86 (C-15), 104.88 (C-19), 98.67 (C-17), 55.83 (C-14, –CH_2_), 55.55 (C-22, –CH_3_), 55.48 (C-21, –CH_3_), 52.54 (C-11, –CH_2_, Piperazine), 52.23 (C-12, –CH_2_, Piperazine), 46.76 (C-13, –CH_2_, Piperazine), 41.56 (C-10, –CH_2_, Piperazine), 28.34 (C-7, –CH_3_). ESI-Mass for C_22_H_26_N_2_O_4_ *m/z*: 383.300 [M + H]^+^.

1–(2-(4–(2-Methoxybenzyl)piperazine-1-carbonyl)phenyl)ethan-1-one (**6n**) white solid; Yield: 40%; m.p. 117.9–119.7 °C. ^1^H NMR (400 MHz, DMSO-*d_6_*) δ 7.98 (dd, *J* = 7.8, 1.3 Hz, 1H, Ar-**H**), 7.63 (dd, *J* = 7.6, 1.3 Hz, 1H, Ar-**H**), 7.55 (dd, *J* = 7.6, 1.3 Hz, 1H, Ar-**H**), 7.35 – 7.20 (*m*, 3H, Ar-**H**), 6.97 (dd, *J* = 8.3, 1.1 Hz, 1H, Ar-**H**), 6.92 (dd, *J* = 8.3, 1.2 Hz, 1H, Ar-**H**), 3.77 (*s*, 3H, OC**H_3_**), 3.59 (*s*, 2H, piperazine-**H**), 3.49 (*s*, 2H, C**H_2_**), 3.05 (*t*, *J* = 5.0 Hz, 2H, piperazine-**H**), 2.55 (*s*, 3H, C**H_3_**), 2.47 (*t*, *J* = 5.0 Hz, 2H, piperazine-**H**), 2.31 (*t*, *J* = 5.0 Hz, 2H, piperazine-**H**). ^13 ^C NMR (100 MHz, DMSO-*d_6_*) δ: 199.23 (C-6, C=O, ketone), 169.36 (C-9, C=O, amide), 157.79 (C-16), 137.12 (C-5), 135.76 (C-8), 132.95 (C-2), 130.31 (C-20), 130.28 (C-3), 129.28 (C-4), 128.58 (C-15), 127.42 (C-1), 125.82 (C-18), 120.57 (C-19), 111.22 (C-17), 55.76 (C-14, –CH_2_; C-21, –CH_3_), 52.69 (C-11, –CH_2_, Piperazine), 52.42 (C-12, –CH_2_, Piperazine), 46.77 (C-13, –CH_2_, Piperazine), 41.57 (C-10, –CH_2_, Piperazine), 28.33 (C-7, –CH_3_). ESI-Mass for C_21_H_24_N_2_O_3_ *m/z*: 353.251 [M + H]^+^.

1–(2-(4–(3-Methoxybenzyl)piperazine-1-carbonyl)phenyl)ethan-1-one (**6o**) white solid; Yield: 45%; m.p. 119.3–120.7 °C. ^1^H NMR (400 MHz, DMSO-*d_6_*) δ: 7.98 (dd, *J* = 7.8, 1.3 Hz, 1H, Ar-**H**), 7.63 (dd, *J* = 7.5, 1.2 Hz, 1H, Ar-**H**), 7.55 (dd, *J* = 7.6, 1.2 Hz, 1H, Ar-**H**), 7.32 – 7.19 (*m*, 2H, Ar-**H**), 6.92 – 6.85 (*m*, 2H, Ar-**H**), 6.85 – 6.78 (*m*, 1H, Ar-**H**), 3.74 (*s*, 3H, OC**H_3_**), 3.58 (*t*, *J* = 5.0 Hz, 2H, C**H_2_**), 3.47 (*s*, 2H, C**H_2_**), 3.05 (*t*, *J* = 5.0 Hz, 2H, piperazine-**H**), 2.55 (*s*, 3H, C**H_3_**), 2.42 (*t*, *J* = 5.0 Hz, 2H, piperazine-**H**), 2.28 (*t*, *J* = 5.0 Hz, 2H, piperazine-**H**); ^13 ^C NMR (100 MHz, DMSO-*d*_6_) δ: 199.22 (C-6, C=O, ketone), 169.35 (C-9, C=O, amide), 158.78 (C-17), 137.12 (C-5), 135.80 (C-8), 132.92 (C-2), 130.54 (C-15), 130.25 (C-3), 130.12 (C-19), 129.28 (C-4), 127.41 (C-1), 125.37 (C-20), 114.17 (C-18), 114.04 (C-16), 61.78 (C-14, –CH_2_), 55.45 (C-21, –CH_3_), 52.47 (C-11, –CH_2_, Piperazine), 52.16 (C-12, –CH_2_, Piperazine), 46.75 (C-13, –CH_2_, Piperazine), 41.54 (C-10, –CH_2_, Piperazine), 28.33 (C-7, –CH_3_). ESI-MS for C_21_H_24_N_2_O_3_ *m/z*: 353.240 [M + H]^+^.

1–(2-(4-(Benzo[d][1,3]dioxol-5-ylmethyl)piperazine-1-carbonyl)phenyl)ethan-1-one (**6p**) white solid; Yield: 36%; m.p. 103.4–104.5 °C. ^1^H NMR (400 MHz, DMSO-*d_6_*) δ 7.98 (dd, *J* = 7.8, 1.3 Hz, 1H, Ar-**H**), 7.63 (dd, *J* = 7.6, 1.2 Hz, 1H, Ar-**H**), 7.55 (dd, *J* = 7.6, 1.2 Hz, 1H, Ar-**H**), 7.28 (dd, *J* = 7.8, 1.3 Hz, 1H, Ar-**H**), 6.92 – 6.80 (*m*, 2H, Ar-**H**), 6.75 (dd, *J* = 7.9, 1.3 Hz, 1H, Ar-**H**), 5.99 (*s*, 2H, OC**H_3_**), 3.58 (*t*, *J* = 5.0 Hz, 2H, piperazine-**H**), 3.40 (*s*, 2H, C**H_2_**), 3.04 (*t*, *J* = 5.0 Hz, 2H, piperazine-**H**), 2.55 (*s*, 3H, C**H_3_**), 2.42 (*t*, *J* = 5.0 Hz, 2H, piperazine-**H**), 2.26 (*t*, *J* = 5.0 Hz, 2H, piperazine-**H**). ^13 ^C NMR (100 MHz, DMSO-*d_6_*) δ: 199.21 (C-6, C=O, ketone), 169.35 (C-9, C=O, amide), 147.68 (C-19), 146.65 (C-18), 137.12 (C-5), 135.74 (C-8), 132.94 (C-2), 132.17 (C-15), 130.28 (C-3), 129.28 (C-4), 127.40 (C-1), 122.44 (C-16), 109.50 (C-17), 108.31 (C-20), 101.24 (C-21), 62.03 (C-14, –CH_2_), 52.42 (C-11, –CH_2_, Piperazine), 52.12 (C-12, –CH_2_, Piperazine), 46.74 (C-13, –CH_2_, Piperazine), 41.52 (C-10, –CH_2_, Piperazine), 28.32 (C-7, –CH_3_). ESI-Mass for C_21_H_22_N_2_O_4_ *m/z*: 367.239 [M + H]^+^.

1–(2-(4-((2,3-Dihydrobenzo[b][1,4]dioxin-6-yl)methyl)piperazine-1-carbonyl)phenyl)ethan-1-one (**6q**) white solid; Yield: 40%; m.p. 124.9–126.4 °C. ^1^H NMR (400 MHz, DMSO-*d_6_*) δ: 7.98 (dd, *J* = 7.8, 1.3 Hz, 1H, Ar-**H**), 7.63 (dd, *J* = 7.6, 1.3 Hz, 1H, Ar-**H**), 7.55 (dd, *J* = 7.6, 1.3 Hz, 1H, Ar-**H**), 7.28 (dd, *J* = 7.8, 1.3 Hz, 1H, Ar-**H**), 6.84 – 6.71 (*m*, 3H, Ar-**H**), 4.21 (*s*, 4H, OC**H_2_**×2), 3.58 (*t*, *J* = 5.0 Hz, 2H, piperazine-**H**), 3.37 (*s*, 2H, C**H_2_**), 3.04 (*t*, *J* = 5.0 Hz, 2H, piperazine-**H**), 2.55 (*s*, 3H, C**H_3_**), 2.41 (*t*, *J* = 5.0 Hz, 2H, piperazine-**H**), 2.26 (*t*, *J* = 5.0 Hz, 2H, piperazine-**H**). ^13 ^C NMR (100 MHz, DMSO-*d_6_*) δ: 199.22 (C-6, C=O, ketone), 169.35 (C-9, C=O, amide), 143.50 (C-19), 142.82 (C-18), 137.14 (C-5), 135.74 (C-8), 132.95 (C-2), 131.31 (C-15), 130.28 (C-3), 129.29 (C-4), 127.40 (C-1), 122.07 (C-16), 117.80 (C-17), 117.13 (C-20), 64.47 (C-22), 64.42 (C-21), 61.76 (C-14, –CH_2_), 52.50 (C-11, –CH_2_, Piperazine), 52.15 (C-12, –CH_2_, Piperazine), 46.74 (C-13, –CH_2_, Piperazine), 41.53 (C-10, –CH_2_, Piperazine), 28.33 (C-7, –CH_3_). ESI-Mass for C_22_H_24_N_2_O_4_ *m/z*: 381.248 [M + H]^+^.

1–(2-(4-((3,5,6-Trimethylpyrazin-2-yl)methyl)piperazine-1-carbonyl)phenyl)ethan-1-one (**6r**) white solid; Yield: 47%; m.p. 119.0–120.1 °C. ^1^H NMR (400 MHz, DMSO-*d_6_*) δ: 7.98 (dd, *J* = 7.8, 1.3 Hz, 1H, Ar-**H**), 7.64 (dd, *J* = 7.5, 1.2 Hz, 1H, Ar-**H**), 7.55 (dd, *J* = 7.5, 1.3 Hz, 1H, Ar-**H**), 7.29 (dd, *J* = 7.8, 1.3 Hz, 1H, Ar-**H**), 3.58 (*s*, 2H, C**H_2_**), 3.55 (*t*, *J* = 5.0 Hz, 2H, piperazine-**H**), 3.01 (*t*, *J* = 5.0 Hz, 2H, piperazine-**H**), 2.55 (*s*, 3H, C**H_3_**), 2.50 (*s*, 3H, C**H_3_**), 2.47 (*t*, *J* = 5.0 Hz, 2H, piperazine-**H**), 2.40 (d, *J* = 4.3 Hz, 6H, C**H3 × 2**), 2.30 (*t*, *J* = 5.0 Hz, 2H, piperazine-**H**). ^13 ^C NMR (100 MHz, DMSO-*d_6_*) δ: 199.22 (C-6, C=O, ketone), 169.36 (C-9, C=O, amide), 149.99 (C-19), 149.88 (C-16), 147.94 (C-18), 147.61 (C-15), 137.08 (C-5), 135.73 (C-8), 132.95 (C-2), 130.28 (C-3), 129.29 (C-4), 127.41 (C-1), 61.78 (C-14, –CH_2_), 52.71 (C-11, –CH_2_, Piperazine), 52.51 (C-12, –CH_2_, Piperazine), 46.71 (C-13, –CH_2_, Piperazine), 41.53 (C-10, –CH_2_, Piperazine), 28.32 (C-7, –CH_3_), 21.54 (C-23), 21.44 (C-22), 20.93 (C-21). ESI-Mass for C_21_H_26_N_4_O_2_ *m/z*: 367.306 [M + H]^+^.

1–(2-(4-(Pyridin-4-ylmethyl)piperazine-1-carbonyl)phenyl)ethan-1-one (**6 s**) white solid; Yield: 44%; m.p. 129.0 – 131.2 °C. ^1^H NMR (400 MHz, DMSO-*d_6_*) δ: 8.55 – 8.47 (*m*, 2H, Py-**H**), 7.98 (dd, *J* = 7.8, 1.3 Hz, 1H, Ar-**H**), 7.63 (dd, *J* = 7.5, 1.3 Hz, 1H, Ar-**H**), 7.55 (dd, *J* = 7.6, 1.3 Hz, 1H, Ar-**H**), 7.38 – 7.32 (*m*, 2H, Py-**H**), 7.29 (dd, *J* = 7.8, 1.3 Hz, 1H, Ar-**H**), 3.62 (*t*, *J* = 5.0 Hz, 2H, piperazine-**H**), 3.54 (*s*, 2H, C**H_2_**), 3.07 (*t*, *J* = 5.0 Hz, 2H, piperazine-**H**), 2.56 (*s*, 3H, C**H_3_**), 2.47 (*t*, *J* = 5.0 Hz, 2H, piperazine-**H**), 2.30 (*t*, *J* = 5.0 Hz, 2H, piperazine-**H**). ^13 ^C NMR (100 MHz, DMSO-*d_6_*) δ: 199.23 (C-6, C=O, ketone), 169.41 (C-9, C=O, amide), 150.02 (C-16), 147.59 (C-18), 137.08 (C-5), 135.72 (C-8), 132.97 (C-2), 130.32 (C-3), 129.31 (C-4), 127.41 (C-1), 124.19 (C-19), 60.90 (C-14, –CH_2_), 52.57 (C-11, –CH_2_, Piperazine), 52.35 (C-12, –CH_2_, Piperazine), 46.70 (C-13, –CH_2_, Piperazine), 41.49 (C-10, –CH_2_, Piperazine), 28.32 (C-7, –CH_3_). ESI-Mass for C_19_H_21_N_3_O_2_ *m/z*: 324.257 [M + H]^+^.

#### 2.1.2. The procedure of synthetic for (4-benzylpiperazin-1-yl) (phenyl) methanone (8)

Benzoic acid **7** (370 mg 3 mmol) was dissolved in DCM and then oxalyl chloride (630 mg, 5 mmol) and DMF (3 drops) were added at 0 °C. After reaction at 25 °C for 2 h, the reaction mixture was concentrated *in vacuo*. The residue in DCM was added dropwise to a solution of 1–(4-chlorbenzyl)piperazine (840 mg, 4.0 mmol) and Et_3_N (610 mg, 6 mmol) at 0 °C. The reaction mixture was stirred at 25 °C for 3 h and concentrated *in vacuo*. To the residue was added DCM and the mixture was washed successively with H_2_O, brine, dried over Na_2_SO_4_ and concentrated *in vacuo*. The residue was purified by flash column chromatography utilising PE/EA (2:1) as the eluent to afford the title compound as white solid; Yield: 64%; m.p. 101.0 – 101.9 °C. ^1^H NMR (400 MHz, DMSO-*d_6_*) δ: 7.48 – 7.42 (*m*, 3H, Ar-**H**), 7.42 – 7.36 (*m*, 4H, Ar-**H**), 7.36 – 7.31 (*m*, 2H, Ar-**H**), 3.62 (*s*, 2H, piperazine-**H**), 3.50 (*s*, 2H, C**H_2_**), 3.36 (*s*, 2H, piperazine-**H**), 2.38 (d, *J* = 26.3 Hz, 4H, piperazine-**H**). ^13 ^C NMR (100 MHz, DMSO-*d_6_*) δ: 169.34 (C-7, C=O, amide), 137.27 (C-6), 136.32 (C-13), 132.04 (C-16), 131.14 (C-3), 129.95 (C-14, 18), 128.86 (C-1, 5), 128.65 (C-14, 17), 127.35 (C-2, 4), 61.31 (C-12, –CH_2_), 53.11 (C-9, –CH_2_, Piperazine), 52.61 (C-10, –CH_2_, Piperazine), 47.58 (C-11, –CH_2_, Piperazine), 41.96 (C-8, –CH_2_, Piperazine). ESI-Mass for C_18_H_19_ClN_2_O *m/z*: 315.196 [M + H]^+^.

### 2.2. Biological evaluation

#### 2.2.1. Animals

Male ICR mice (20 ± 2 g) were obtained from the Experimental Animal Centre of Anhui Medical University (Hefei, China). The mice reared in a pathogen-free setting (23 ± 2 °C, 55 ± 5% humidity) with free access to water and pelleted food throughout the experimental cycle. All experimental procedures performed in accordance with guide for the Care and Use of Laboratory Animals (National Research Council, 1996) and were approved by the Experimental Animal Ethics Committee of Anhui University of Chinese Medicine (Hefei, China).

#### 2.2.2. DNFB-induced mouse model of ACD

After 7 days acclimatisation, the mice were randomly divided into control group, ACD model group, dexamethasone positive control group and 20 compound groups, six in each group. The control group and the model group were given the same dose of the vehicle, the positive control group was given dexamethasone 0.5 mg/kg, and the test compound groups were given compound 5 mg/kg, all of which were given by intragastric administration and were administered once a day for 6 days. Except for the control group, the 50 µL of 1% DNFB (in acetone/olive oil 4:1) was administered to the stripped epidermis, and repeated the next day. After 6 days of drug treatment, 10 µL of 1% DNFB acetone olive oil solution was applied on both sides of the right ear to stimulate inflammation, and equivalent acetone olive oil solution was applied on the left ear for comparison. After 24 h, mice were sacrificed, ears were cut off along the baseline of both ears, and round ear slices were sheared in the same part of both ears with a 6 mm hole puncher and calculate the swelling degree and inhibition rate after weighing. Calculation was carried out according to the following equations.

Swelling degree (mg) = MR – ML

Inhibitory rate of ear swelling (%)=((MR – ML) model – (MR – ML) treated)/(MR – ML) model   ×100%

MR=Average weight of the right ear slices;ML= Average weight of the left ear slices

#### 2.2.3. Cell culture

Murine RAW 264.7 macrophages were obtained from the American Type Culture Collection (ATCC, USA). The cells were incubated in DMEM media supplemented with 10% FBS, 100 U/mL penicillin and 100 mg/mL streptomycin at 37 °C with 5% CO_2_.

#### 2.2.4. Cell viability assay

Cell viability was assessed using the MTT assay. RAW 264.7 cells were inoculated in 96-well plate at a density of 1.0 × 10^5^ cells per well. After incubated for 24 h, the cells were treated with various concentrations of the compound. After an additional 24-h incubation, 20 µL of 0.5 mg/mL MTT reagent was added to wells and incubated for another 4 h. After 4 h, cell culture media was removed and DMOS was added into each well, and then, the optical density was measured at 570 nm. The IC_50_ values were determined by GraphPad Prism 6.

#### 2.2.5. Measurement of NO production

The RAW 264.7 macrophages were inoculated at 96-well plates and were pre-treated with vehicle or **6n** (0–20 µM dose range) for 2 h and then stimulated with LPS (200 ng/mL) for 24 h. NO concentration in the medium was determined using Griess reagent kit (Beyotime, China) at 540 nm with a microplate reader (MQX200, Bio-Tek, USA) and calculated the inhibition rate of NO.

#### 2.2.6. Western blot

The cell in 96-well plates were treated as described above and then stimulated with LPS (200 ng/mL) for 24 h. The cells were harvested and lysed in an extraction lysis buffer (Beyotime Biotechnology, Shanghai, China) containing protease inhibitors. The protein concentration was determined using a BCA protein assay kit (Thermo Scientific, 23227). The whole cell lysates were separated by 10% sodium dodecyl sulphate-polyacrylamide gel electrophoresis and transferred to a nitrocellulose membrane. Each membrane was incubated with Tris-buffered saline (pH 7.6, containing 0.05% Tween-20 and 5% non-fat milk). The nitrocellulose membrane was incubated with the primary antibody against p-JNK1/2, JNK1/2, p-p38, p38, p-ERK1/2, ERK1/2, IκBa, NF-κB p65, COX-2, iNOS or β-Actin (Abcam, Cambridge, UK). Immunoreactive bands were detected by incubating with horseradish peroxidase-conjugated secondary antibodies, and visualised using enhanced chemiluminescence reagents (Bio-Rad, Hercules, CA).

#### 2.2.7. p38α MAPK inhibition assay *in vitro*

The *in vitro* ability of test compounds and SB203580 (PerkinElmer, Boston, MA, USA) to inhibit p38α MAPK were measured according to the method reported by Babu J. Mavunkel^16^. In brief, after mixing the enzyme reagent with the sample, a reaction mixture containing 200 µM biotin-peptide substrate and 600 µM ATP (+100 µCi/mL γ-^32 ^P-ATP) was added to initiate the reaction. After incubation at 30 °C for 60 min, 10 µL of 1.5% phosphoric acid solution was added to terminate the reaction. Part of the reaction solution was transferred to the well of a streptavidin-coated flash plate, washed in PBS containing 0.01% Tween and sealed. The average value of counts per minute for each group and the IC_50_ value was calculated.

The average fluorescence values of each well were calculated and recorded as RFU (Relative Fluorescence Unit) blank control (RFU blank), RFU 100% enzyme activity control (RFU enzyme), RFU positive drug control (RFU drug) and RFU test compound (RFU compound). The inhibition rate is calculated according to the following formula.

Inhibition rate (%)= (RFU (enzyme) – RFU (compound/drug) – RFU (blank))     /(RFU (enzyme) – RFU (blank)) * 100%

The IC_50_ value of the test compound was calculated by the concentration-inhibition reaction curve and the assays were performed in triplicate.

#### 2.2.8. COX-1 and COX-2 inhibition assay *in vitro*

The COX-1/COX-2 inhibitory activity of test compounds and celecoxib were determined by COX Inhibitor Screening Kit (Fluorometric) (BioVision, Inc., Mountain View, CA, USA) according to the manufacturer's instructions. Simply, different concentrations of the test compound solution were added to the mixed solution containing COX-1/COX-2 enzyme (10 µL) and Assay Buffer (960 µL, 0.1 M Tris-HCl pH 8.0 containing 5 µM EDTA and 2 µM phenol). After the addition of the arachidonic acid solution (100 µM), the mixture was kept at 37 °C in the dark for 5 min and then added 50 µL of 1 M HCl to stop the reaction. The fluorescence value was measured with an excitation wavelength of 535 nm and an emission wavelength of 587 nm. The IC_50_ values were calculated as described above.

### 2.3. Molecular docking

The X-ray crystal structure of p38α MAPK (PDB code: 2QD9), COX-1 (PDB code: 1PGF) and COX-2 (PDB code: 1CX2) were obtained from Protein Data Bank. Before docking, the 3 D structures of **6n** was generated and the energy minimisation was carried out; removing water moleculars and adding hydrogen atoms to p38α MAPK, COX-1 and COX-2 with the AutoDock Tools^17^. Then, the docking was performed by Autodock 4.2 programme with Lamarckian genetic algorithm to sift the best ligand enzyme interaction. The final graphical representations were rendered by PyMOL^18^.

**Figure 3.**
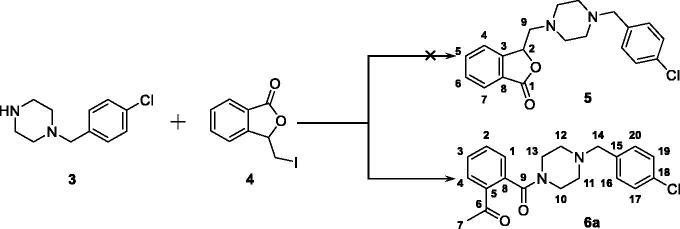
The generation of compound **6a**, an unexpected product, from the reaction between **3** and **4**.

## 3. Result and discussion

### 3.1. Chemistry

In our attempt to prepare the 3-butylphthalide derivative **5** via the nucleophilic substitution between 1–(4-chlorobenzyl)piperazine **3** and 3-(iodomethyl)isobenzofuran-1 (3*H*)-one **4**, an unexpected main product was afforded. Despite its MS consistent with the chemical structure of **5**, no characteristic signal of 2-CH appeared in the ^1^H NMR spectrum, while a singlet with the integral of 3 existed at 2.56 ppm. Meanwhile, two signals appeared at 170.28 and 198.57 ppm, respectively, in the ^13 ^C NMR spectrum, indicating the existence of two carbonyl groups in the chemical structure of the product. Taken together, it was speculated that the afforded product was **6a** (Figure 3). The singlet at 2.56 ppm was generated from its acetyl moiety.

**Figure 4.**
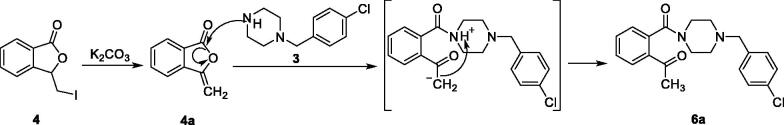
The possible mechanism for the generation of **6a**.

The speculated mechanism for the generation of **6a** was displayed in Figure 4. Intermediate **4** firstly underwent elimination to give enol ester **4a**. Afterwards, the five-membered lactone was opened in the presence of 1–(4-chlorobenzyl)piperazine. Finally, the proton on the piperazine was transferred to the carbonyl alpha site to form compound **6a**.

**Figure 5.**
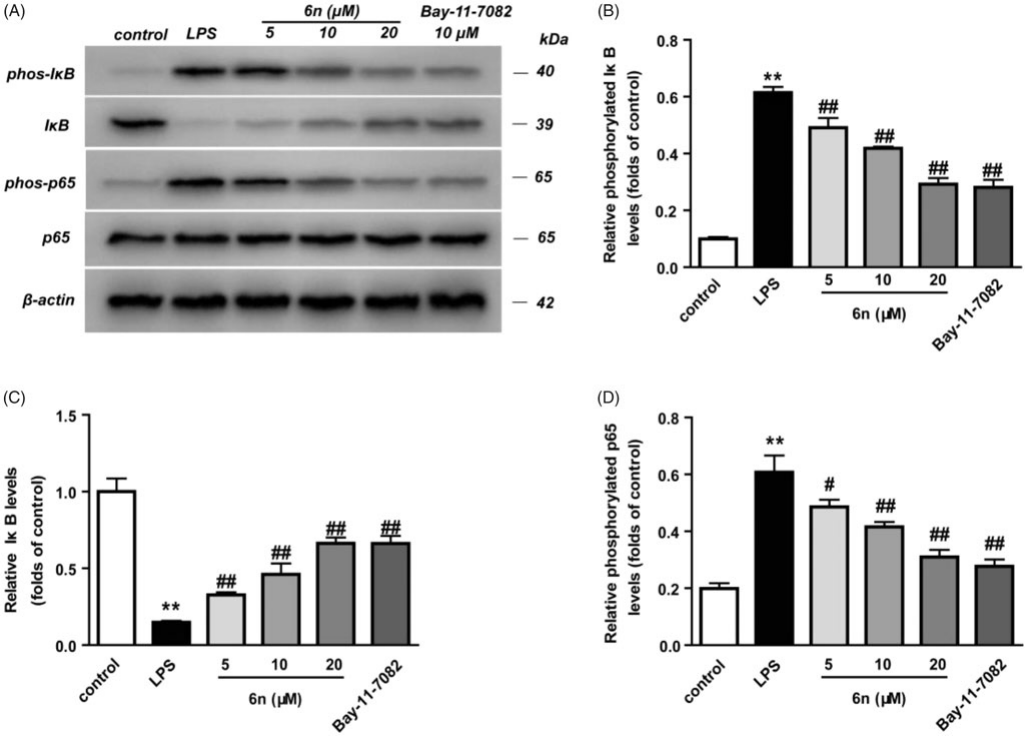
Compound **6n** inhibited LPS-induced NF-κB activation in RAW264.7 cells. RAW264.7 cells were co-incubated with **6n** (5, 10, 20 μM) and LPS (200 ng/mL) for 30 min. The levels of NF-κB p65, IκB, and their phosphorylated forms were analysed using western blot. The results were showed as means ± SD (*n* = 3); **p < 0.01 vs. compared with the control group; ^#^*p* < 0.05, ^##^*p* < 0.01 vs. compare with LPS-stimulated group.

**Scheme 1.**
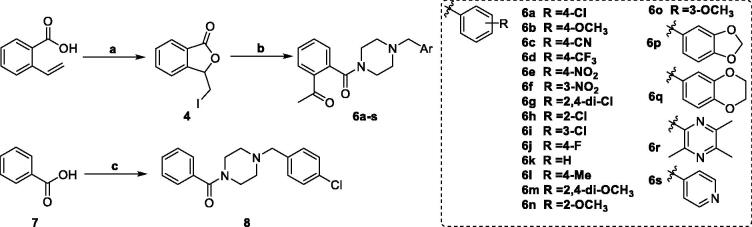
Reagents and conditions: (a) I_2_, CH_3_CN, 25 °C; (b) K_2_CO_3_, CH_3_CN, 25 °C; (c) oxalyl chloride, 1–(4-chlorbenzyl)piperazine, DCM, Et_3_N, 25 °C.

The synthetic route to target talmapimod analogues **6a-s** and **8** was displayed in Scheme 1. 3-(Iodomethyl)isobenzofuran-1 (3*H*)-one **4** was prepared according to the procedure reported by Siegfried H. Reich *et al.*^19^. It was treated with a series of benzyl piperazine derivatives in the presence of K_2_CO_3_ to furnish compounds **6a-s**. After converting benzoic acid **7** into benzoyl chloride, it was condensed with 1–(4-chlorobenzyl)piperazine **3** to afford **8** as the product.

### 3.2. Biological evaluation

#### 3.2.1. Anti-inflammatory activity *in vivo*

To validate their anti-inflammatory efficacy, we evaluated compounds **6a-s** and **8** *in vivo* at a p.o. dose of 5 mg/kg in a 2,4-dinitrofluorobenzenethe-induced (DNFB-induced) mouse model of allergic contact dermatitis^20^. Dexamethasone (DEX) at a p.o. dose of 0.5 mg/kg was employed as the positive control. After the mice were sacrificed, the swelling degree and inhibition rate were calculated by weighing the same part of both ears. The results demonstrated that compounds **6f**, **6j**, **6n**, **6p** and **8** had significant inhibitory activity (*p* < 0.01). In particular, **6n** exerted the strongest *in vivo* anti-inflammatory activity with the inhibition rate of 46.3%. Hence, **6n** was further selected for exploring the molecular mechanisms underlying its anti-inflammatory efficacy (Table 1).

**Table 1.** Results of anti-inflammatory activity *in vivo* of compounds and DEX (Mean ± SD, *n* = 6).

Compound	Dose (mg/kg)	Swelling degree (mg)	Inhibition (%)	Compound	Dose (mg/kg)	Swelling degree (mg)	Inhibition (%)
Control	–	–	–	**6j**	5	10.4 ± 3.1^▲▲^	36.6
Model	–	16.4 ± 3.6^△△^	–	**6k**	5	13.0 ± 3.5	20.7
DEX	0.5	7.9 ± 3.0^▲▲^	51.8	**6l**	5	11.4 ± 3.2^▲^	30.5
**6a**	5	11.9 ± 3.7^▲^	27.4	**6m**	5	11.6 ± 2.4^▲^	29.3
**6b**	5	12.0 ± 3.4^▲^	26.8	**6n**	5	8.8 ± 3.2^▲▲^	46.3
**6c**	5	11.3 ± 4.0^▲^	31.1	**6o**	5	13.0 ± 4.4	20.7
**6d**	5	12.5 ± 3.4^▲^	23.8	**6p**	5	11.0 ± 3.2^▲▲^	32.9
**6e**	5	13.0 ± 3.3	20.7	**6q**	5	13.3 ± 2.6	18.9
**6f**	5	9.9 ± 2.9^▲▲^	39.6	**6r**	5	10.8 ± 3.5^▲▲^	34.1
**6g**	5	12.9 ± 2.6^▲^	21.3	**6s**	5	12.9 ± 3.4	21.3
**6h**	5	12.8 ± 4.1	21.9	**8**	5	10.1 ± 2.8^▲▲^	38.4
**6i**	5	12.0 ± 3.0^▲^	26.8				

^△^*p* < 0.05, ^△△^*p* < 0.01 vs control; ^▲^*p* < 0.05, ^▲▲^*p* < 0.01 vs model.

swelling degree (mg) = MR − ML.

inhibition (%) = ((MR − ML) model−(MR − ML) treated)/(MR − ML) model * 100%.

MR: Average weight of the right ear; ML: Average weight of the left ear.

#### 3.2.2. Compound 6n inhibited LPS-induced inflammatory mediators in RAW264.7 cells

**Figure 6.**
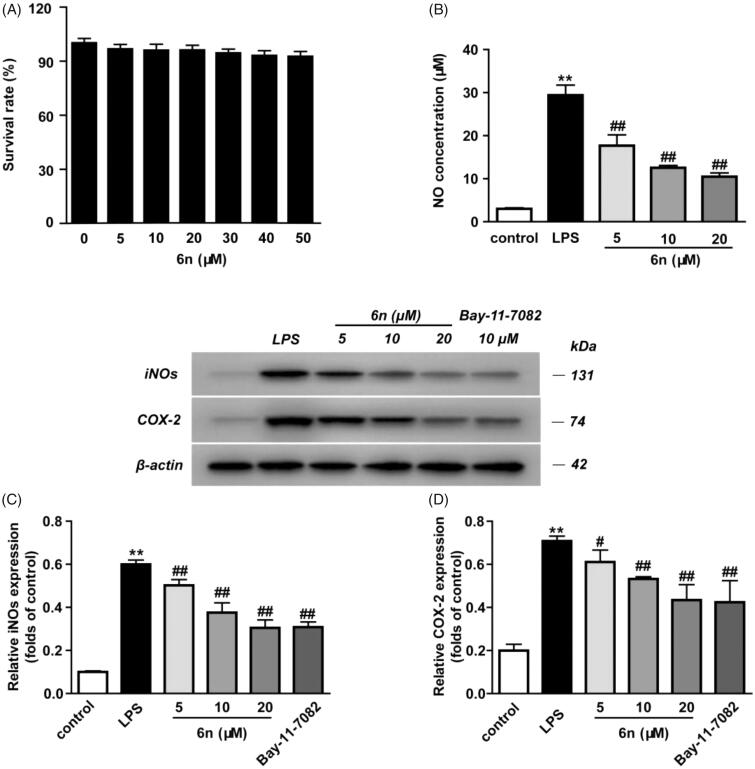
Compound **6n** inhibited LPS-induced iNOS and COX-2 expressions in RAW264.7 cells. The cells were pre-treated with different concentrations of **6n** and then were stimulated with LPS (200 ng/mL) for 24 h. Bay 11–7082 is the NF-κB inhibitor. Cell viability was evaluated using the MTT assay. NO production was measured using nitrite and nitrate assay. iNOS and COX-2 expression were detected by Western blot. (A) Cell viability assay; (B) Quantitative analysis of NO productions; (C) Quantitative analysis of iNOS expressions, (D) Quantitative analysis of COX-2 expressions. β-actin was used as loading control. The results were showed as means ± SD (*n* = 3); **p < 0.01 vs compared with the control group; ^#^*p* < 0.05, ^##^*p* < 0.01 vs compared with LPS-stimulated group.

NO serves as an important inflammation mediator, and its continuous high concentration is involved with the development of inflammation-related diseases^21^. Besides, NO regulates inducible nitric oxide synthetase (iNOS) and cyclooxygenase 2 (COX-2)^22^. In view of these, we examined the inhibitory effect of **6n** on LPS-induced NO production and LPS-induced expressions of iNOS and COX-2 in RAW264.7 cells (Figure 5). The cytotoxicity of **6n** was firstly evaluated, and no significant toxicity was observed at concentrations ranging from 5 to 50 µM (Figures 5 and 6(A). While the LPS (200 ng/mL) stimulation for 24 h significantly increased NO production, compound **6n** reduced LPS-induced NO production in a dose-dependent manner (Figure 6(B)). Besides, **6n**-treatment culminated in a dose-dependent decrease in the LPS-induced expressions of iNOS (Figure 6(C)) and COX-2 (Figure 6(D)).

#### 3.2.3. Compound 6n inhibited LPS-induced nuclear factor-kappa B (NF-κB) activation in RAW264.7 cells

The activation of NF-κB through proteasomal degradation and phosphorylation of inhibitory κB (IκB) led to the translocation of NF-κB p65 and its interaction with the gene promoter region in nucleus, thereby promoting the expression of iNOS and COX-2^3^^,^^23^. Thus, western blot was performed to examine the effect of **6n** on LPS-induced transcriptional activity of NF-κB in RAW264.7 cells. As shown in Figure 5, 2-h pre-treatment with **6n** or Bay 11e7082 before LPS stimulation markedly decreased LPS-induced IκB phosphorylation (Figure 5(B)) and increased cytosolic IκB (Figure 5(C)). Meanwhile, the accumulation of NF-κB p65 subunit in the nucleus was also lowered (Figure 5(D)). These results indicated that **6n** exerted anti-inflammatory effects through modulation of NF-κB-signalling pathway.

#### 3.2.4. Compound 6n inhibited LPS-induced p38 MAPK-signalling activation in RAW264.7 cells

**Figure 7.**
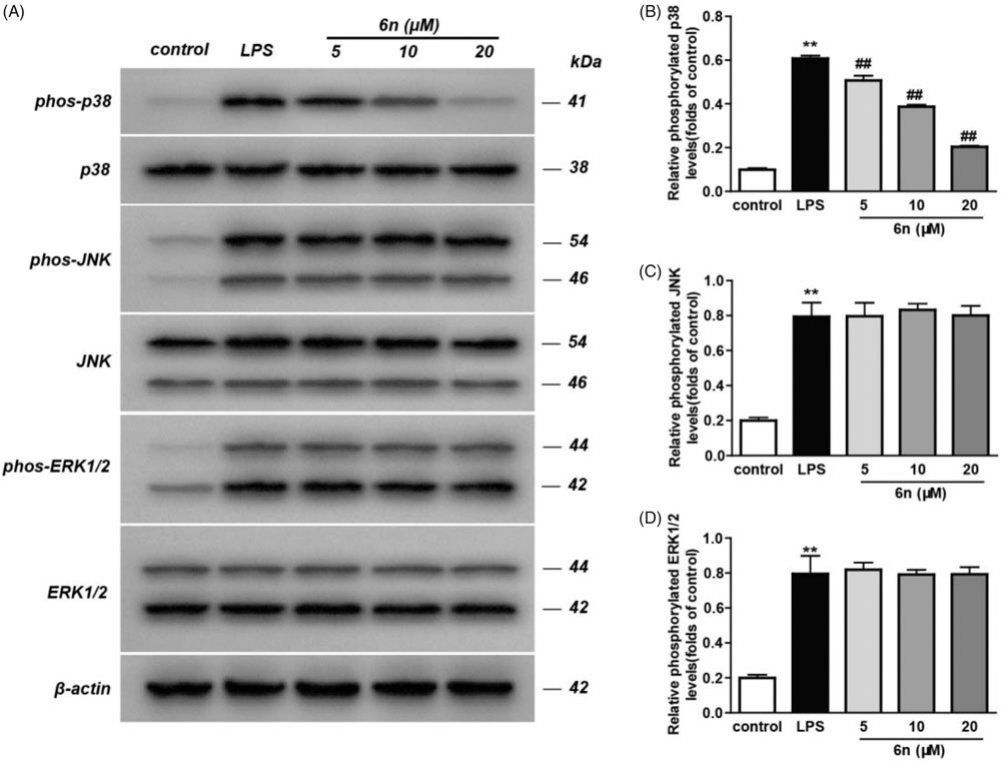
Compound **6n** inhibited LPS-induced MAPK-signalling activation in RAW264.7 cells. RAW264.7 cells were co-incubated with **6n** (5, 10, 20 μM) and LPS (200 ng/mL) for 30 min. The levels of p38, JNK, ERK1/2 and their phosphorylated forms were analysed using western blot. The results were showed as means ± SD (*n* = 3); **p < 0.01 vs compared with the control group; ^#^*p* < 0.05, ^##^*p* < 0.01 vs compare with LPS-stimulated group.

MAPKs play pivotal roles in incurring the immune-mediated inflammation by regulating the transcription and translation of a variety of crucial transcription factors, including activation protein-1 (AP-1) and NF-κB^24^. Inflammatory stimuli trigger a signalling cascade mediated by p38 MAPK, which activates transcription and translation of genes associated with inflammatory responses such as *TNF-α*, *IL-1β* and *IL-6*, and further induces the expression of inflammatory mediators such as COX-2, iNOS and adhesion molecules^25^. Considering this, we examined the impact of **6n** on the phosphorylation of the three MAPK subtypes, including p38, JNK and ERK. As a result, **6n** can significantly lower p38 phosphorylation (Figure 7(B)), though there was no obvious effect on the phosphorylation of JNK (Figure 7(C)) and ERK (Figure 7(D)). These results suggested that the anti-inflammatory effect of **6n** was related to the inhibition of p38 phosphorylation.

#### 3.2.5. Anti-inflammatory activity *in vitro*

The down-regulation of p38 MAPK phosphorylation by **6n** may be attributed to its interaction with the upstream effector. Besides, owing to the structural similarity of the target compounds to talmapimod, their inhibition against p38α MAPK was expected. This concurrent inhibition of p38α MAPK and its upstream effector would contribute to a two-spot ablation of p38α MAPK-related signalling pathway, which might be beneficial to anti-inflammatory treatment. Thereby, **6f**, **6j**, **6n** and **8**, with strong anti-inflammatory activity, were further evaluated against p38α MAPK with SB203580, a selective p38α MAPK inhibitor, as the reference. Besides, we also evaluated them against COX-2, a well-established anti-inflammatory target, given the correlation of COX-2 with MAPK signalling. The results demonstrated that **6n** exhibited attractive inhibitory activity against p38α MAPK with IC_50_ value of 1.95 µM, along with potent inhibitory activity against COX-2 with IC_50_ value of 0.036 µM, which was comparable to that of Celecoxib. Besides, it inhibited COX-2 with a favourable selectivity, which was beneficial to lowering gastrointestinal intolerance. The concomitant inhibition of p38α MAPK, its upstream effector, as well as COX-2 may account for the most favourable anti-inflammatory activity of **6n**. In addition, compound **6j** was identified as a potent inhibitor against COX-2 with IC_50_ value of 0.022 µM, while compound **8** was characterised as a potent inhibitor against p38α MAPK with IC_50_ value of 1.05 µM (Table 2).

**Table 2.** P38α MAPK, COX-1 and COX-2 inhibition activity *in vitro*.

Compound	IC_50_^a^ (mpou)			
	p38p	COX-1	COX-2	SI^b^ (COX-1/COX-2)
**6f**	3.41 ± 0.22	1.19 ± 0.08	0.842 ± 0.062	1.41
**6j**	8.49 ± 0.57	4.23 ± 0.23	0.022 ± 0.003	192.27
**6n**	1.95 ± 0.13	12.59 ± 0.79	0.036 ± 0.004	349.72
**8**	1.05 ± 0.09	6.69 ± 0.41	0.244 ± 0.013	27.42
SB203580	0.39 ± 0.02	ND	ND	ND
Celecoxib	ND	14.51 ± 0.86	0.015 ± 0.001	967.33

ND: not determined.

^a^ IC_50_: Represents the concentration of the test compound that is required for 50% inhibition *in vitro*.

^b^ SI: IC_50_ (COX-1)/IC_50_ (COX-2).

### 3.3. Molecular docking study

The molecular docking analysis of **6f**, **6j**, **6n** and **8** was carried out to elucidate their anti-inflammatory activity *in vitro*. As showed in Table 3, **8** exhibited the lowest binding energy value (−8.98 kcal/mol) when they docked with p38α MAPK, and **6f** bound to COX-2 active site with best binding energy value of −7.82 kcal/mol. This was consistent with the above-mentioned *in vitro* enzymatic experiment results.

**Table 3.** Docking results of **6f**, **6j**, **6n** and **8**

Compound	Energy value (kcal/mol)	
	p38r	COX-2
**6f**	−8.80	−7.55
**6j**	−8.66	−7.82
**6n**	−8.89	−7.72
**8**	−8.98	−7.66

**Figure 8.**
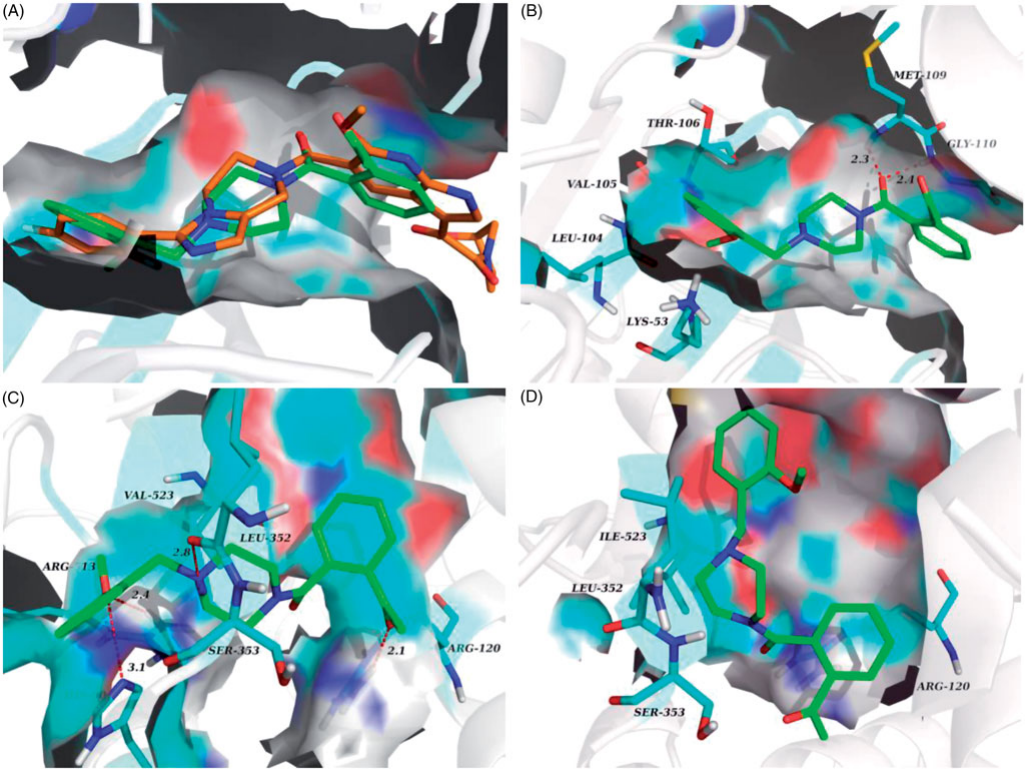
(A) Overlay of docking poses of **6n** (green sticks) and the co-crystallised ligand (orange sticks) in the binding site of p38α MAPK. (B) Docking and binding pattern of **6n** (green sticks) into p38α MAPK active site. (C) Docking and binding pattern of **6n** (green sticks) into COX-2 active site. (D) Docking and binding pattern of **6n** (green sticks) into COX-1 active site. Dashed lines represent hydrogen bonds.

The prominent inhibitory activity against p38α MAPK and COX-2 contributed to the most potent *in vivo* anti-inflammatory activity of **6n**. It bound to the p38α MAPK active site in a similar manner to the co-crystallized ligand (Figure 8(A)), with the amide carbonyl engaged in H-bond contacts with residues Met 109 and Gly 110 in the hinge region, and 2-methoxybenzyl projected into the hydrophobic pocket formed by Thr 106, Val 105, Leu 104 and Lys 53 (Figure 8(B)). Moreover, the molecular docking results also accounted for the potency of **6n** against COX-2 and its COX-2 selectivity. In compared with COX-1, the active site of COX-2 featured an additional side pocket surrounded by Val 523, Leu 352 and Ser 353^14^^,^^26–28^. The 2-methoxybenzyl moiety of **6n** was embedded in this pocket, with the methoxyl group forming two critical H-bonds with Arg 513 and His 90, and the piperazine nitrogen tethered to the 2-methoxybenzyl moiety formed a H-bond with Lue 352 at the mouth of the pocket. Furthermore, the acetyl on benzene ring was located at the other end of the active site and was involved with H-bond contact with Arg 120 (Figure 8(C)). Interestingly, due to the lack of the side pocket, the binding poses of **6n** in the COX-1 active site was forced to rotate and the above binding interactions was also absent (Figure 8(D)).

**Figure 9.**
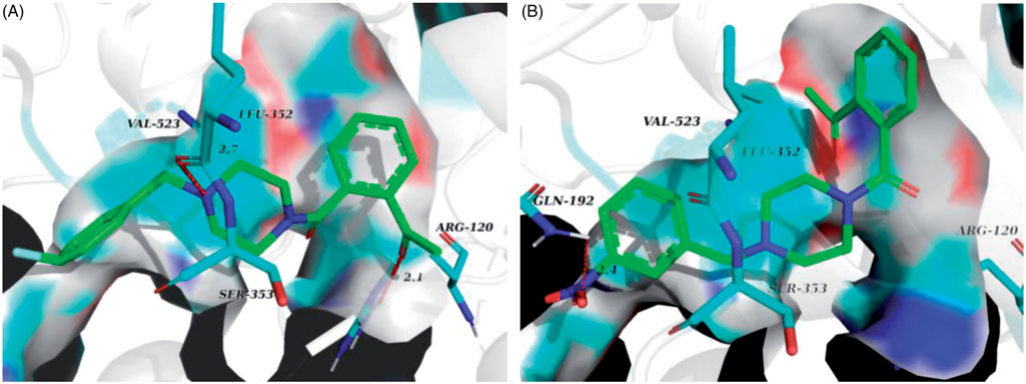
(A) Docking and binding pattern of **6j** (green sticks) into COX-2 active site. (B) Docking and binding pattern of **6f** (green sticks) into COX-2 active site. Dashed lines represent hydrogen bonds.

In comparison to compound **6n**, **6j** bound more strongly to the pivotal residue, that is, Leu 352 (Figure 9(A)). This may be the reason why **6j** had lower binging energy value and better inhibitory activity against COX-2. Loss of two critical H-bonds with Leu 352 and Arg 120, the binding poses of **6f** in COX-2 active site was reversed (Figure 9(B)), and it presented the lowest binging energy value (−7.55 kcal/mol) to other analogues. Correspondingly, the inhibitory activity of **6f** against COX-2 was declining substantially. The result of hydrogen boding analyses may justify that the H-bonds with Leu 352 and Arg 120 were decisive factor for presence of COX-2 inhibitory activity in this series of analogues.

## 4. Conclusions

**Figure 10.**
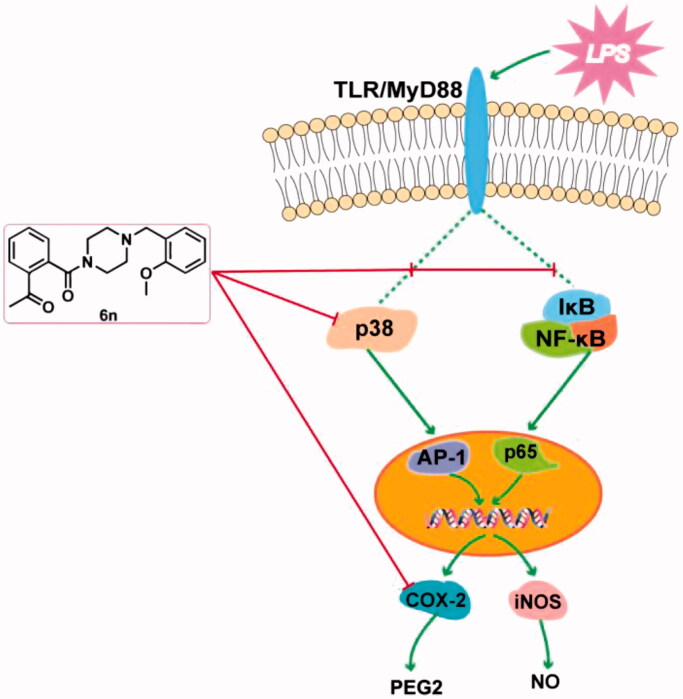
Anti-inflammatory molecular mechanism of **6n**.

In conclusion, we have designed and synthesised a series of talmapimod analogues as the anti-inflammatory agents based on an unexpected product **6a** obtained from an internal programme to prepare butylphthalide derivatives. Among these compounds, **6n** exerted the best anti-inflammatory activity *in vivo*. As illustrated by the mechanism study, **6n**-treatment culminated in a dose-dependent decrease in the LPS-induced expressions of iNOS and COX-2. Besides, **6n**-treatment led to the dose-dependent down-regulations of NF-κB signalling pathway and the p38 MAPK phosphorylation, both of which may contribute to the decrease in LPS-induced expressions of iNOS and COX-2. The down-regulation of p38 MAPK phosphorylation indicated the inhibition of the upstream effector of p38 MAPK. Further *in vitro* enzymatic experiment identified **6n** as a potent inhibitor against both p38α MAPK and COX-2. To our knowledge, this has been the first compound reported to exert p38α MAPK and COX-2 inhibitory activities. Importantly, the concomitant inhibition of p38α MAPK, its upstream effector, and COX-2, along with its confirmed capability to down-regulate NF-κB and MAPK-signalling pathways make **6n** a promising polypharmacological anti-inflammatory agent (Figure 10). The further investigation of **6n** has been currently underway.
